# Expression of *Gallus* Epidermal Growth Factor (gEGF) with Food-Grade *Lactococcus lactis* Expression System and Its Biological Effects on Broiler Chickens

**DOI:** 10.3390/biom11010103

**Published:** 2021-01-14

**Authors:** Yu Zhou, Pinpin Chen, Shuai Shi, Xiaowen Li, Deshi Shi, Zutao Zhou, Zili Li, Yuncai Xiao

**Affiliations:** 1 State Key Laboratory of Agricultural Microbiology, College of Veterinary Medicine, Huazhong Agricultural University, Wuhan 430070, China; ZhouYu2016@webmail.hzau.edu.cn (Y.Z.); starrynight@webmail.hzau.edu (P.C.); sherrylee@webmail.hzau.edu.cn (S.S.); wendy123@webmail.hzau.edu.cn (X.L.); rock@mail.hzau.edu.cn (D.S.); ztzhou@mail.hzau.edu.cn (Z.Z.); lizili@mail.hzau.edu.cn (Z.L.); 2 Key Laboratory of Preventive Veterinary Medicine in Hubei Province, College of Veterinary Medicine, Huazhong Agricultural University, Wuhan 430070, China

**Keywords:** *gallus* epidermal growth factor, *Lactococcus lactis*, growth performance, immune function, intestinal development, broiler chickens

## Abstract

As a multifunctional polypeptide, epidermal growth factor (EGF) increases growth performance or enhances resistance to diseases in commercial broilers under adverse conditions. In this study, a recombinant *Lactococcus lactis* was established to produce the secretory form of bioactive gEGF. The results of in vitro testing showed that gEGF promoted the proliferation of chicken embryo fibroblast cells. A total of 63 5-day-old broiler chickens were evenly divided into three groups and treated with either M17 medium (the control group), supernatant of LL-pNZ8149 fermentation product (the P-LL group), or supernatant of LL-pNZ8149-gEGF fermentation product (the gEGF group). In two weeks, many measurements of growth, immunity and the intestines were significantly higher in the gEGF group than those in the control and the P-LL groups. Our study showed that the bioactive gEGF could be expressed with *Lactococcus lactis* expression system with the potential to enhance growth performance, immune function, and intestinal development in broiler chickens.

## 1. Introduction

The epidermal growth factor (EGF) is a 53-amino acid single-chain polypeptide. EGF is synthesized in the salivary glands and kidneys and, to a lesser extent, in the lactating mammary glands, small intestine, liver, and pancreas [1,2,3]. EGF is an influential molecule involved in multiple biological processes. For example, EGF promotes cell proliferation and differentiation in mice [4]. EGF has also been shown to play important roles in gastrointestinal repair [5], gastric acid secretion [6], and the regulation of both the embryo development [7,8] and the ion transport [9,10]. Recent studies have shown that both the EGF and the epidermal growth factor receptor (EGFR) are involved in tumorigenesis [11,12], while the EGFR has been utilized as a target for cancer therapy [11,12]. Epidermal growth factor (EGF) is a small polypeptide which is produced using genetic techniques, such as expression in *E. coli* [13,14], *Bacillus brevis* [15,16], or *Pichia pastoris* [17,18].

The biological effects of EGF on animals has been widely investigated. The growth performance of early-weaned pigs was enhanced by feeding with a fermentation product of EGF-expressing *Lactococcus lactis* [19]. Dietary supplementation with porcine epidermal growth factor (pEGF) enhanced daily weight gain for 0‒7 days postweaning, significantly increased the IgA serum levels at day 18 postweaning, and significantly increased both the mucosal IgA levels and the crypt depth in the jejunum at day 28 postweaning, indicating that EGF can promote growth performance and immune function in piglets [20]. Furthermore, piglet diets supplemented with EGF can enhance the protection against intestinal pathogens [21,22] and promote the intestinal repair after rotavirus infection [23]. pEGF can also improve the growth prior to the *Eimeria* invasion and improve gut function indices after the invasion in broiler chickens [24]. Rabbit anti-mouse EGF (anti-mEGF) antiserum was administered to pregnant mice from days 10 to 17 during late gestation. Control mice were administered either normal rabbit serum (NRS) or physiological saline (PS). One day prior to birth, the fetuses were removed for the collection of lung samples. This experiment found EGF promotes epithelial cell differentiation of the fetal lung [25].

To date, very few studies have focused on gallus epidermal growth factor (gEGF) and its biological activities. In the current Chinese market, it is imperative to improve the growth performance of young commercial broilers without the treatment of antibiotics, as these drugs have recently been banned from production. Furthermore, it is also important to improve the resilience of chickens under adverse conditions, such as high temperature or stress. Positive effects of EGF have been documented in early-weaned piglets [19] and rats [5]; therefore, it is naturally important to investigate the biological effects of gEGF on chickens. In this study, a recombinant strain of *Lactococcus lactis* (i.e., LL-pNZ8149-gEGF) secreting gEGF was constructed. In order to avoid the risk of using the genetically modified organisms, instead of the direct use of recombinant *Lactococcus lactis*, the supernatant of the LL-pNZ8149-gEGF fermentation product was applied to treat the broilers, with the growth performance, immune function, and intestinal development of these broilers assessed. This study sets the important scientific foundation for the application of gEGF in broiler productivity.

## 2. Materials and Methods

### 2.1. Bacterial Strains, Plasmids, and Medium for Bacterial Growth

*Lactococcus lactis* NZ3900 (NIZO Food Research B.V., Ede, The Netherlands) was cultured in M17 medium (Qingdao Hope Bio-Technology Co., Ltd., Wuhan, China) supplemented with 0.5% (wt/vol) glucose at 30 °C without vibration. The plasmid pNZ8149 was obtained from NIZO Food Research B.V., The Netherlands. *Lactococcus lactis* NZ3900 and the plasmid pNZ8149 were procured from commercial sources. Transformed *L. lactis* cells were selected on M17 medium without glucose.

### 2.2. Construction of the Recombinant Plasmid pNZ8149-gEGF and Transformation of Lactococcus Lactis

The sequence of the mature gEGF peptide was deduced by aligning the amino acid sequence of the pro-gEGF (NCBI Reference Sequence: NP_001001292.1) with that of the mature EGF of other species using Clustal Omega (https://www.ebi.ac.uk/Tools/msa/clustalo/) and the general sequence of EGF-like molecules referred to previously [26]. A codon-optimized fusion gene fragment of gEGF and SP_usp45_ carrying the NcoI/SacI restriction sites and a 6× His-tag was synthesized by AuGCT Co., Ltd. (Wuhan, China), consisting of 287 base pairs (Appendix A). The synthesized gene was digested with NcoI/SacI restriction enzymes (Thermo Fisher Scientific, Waltham, MA, USA) and inserted into the digested pNZ8149 to construct the recombinant plasmid pNZ8149-gEGF. The transformation of *Lactococcus lactis* was performed by electroporation at 2.0 kV for 4.0 ms using a Micropulser (Bio-Rad, Hercules, CA, USA), generating the *L. lactis* strain that produced gEGF (LL-pNZ8149-gEGF). The recombinant plasmid was verified by PCR with the upstream primer pNZ8149-F (5’-GATTTCGTTCGAAGGAACTAC-3’) and the downstream primer pNZ8149-R (5’-ATCAATCAAAGCAACACGTGC-3’) and by restriction enzyme digestion, with the cloned fragments verified by sequencing using primers pNZ8149-F and pNZ8149-R (Tianyi Huiyuan Co., Ltd., Wuhan, China).

### 2.3. Expression of Recombinant gEGF Protein in Lactococcus Lactis

The LL-pNZ8149-gEGF strain was inoculated into 5 mL fresh M17 medium (1:25 dilution). When the optical density at 600 nm (OD_600_) of the bacterial cultures reached 0.4, the expression of gEGF-His6 fusion protein (gEGF) was induced by adding 10 ng/mL nisin (Sigma-Aldrich Co., Ltd., St Louis, MO, USA). The culture was incubated at 37 °C without vibration for 6 h. The presence of the target protein derived from the LL-pNZ8149-gEGF fermentation was verified by its hybridization with the His-tag monoclonal antibody (Abbkine Scientific Co., Ltd., Wuhan, China). To investigate the optimal conditions required for induction, the recombinant strain of *L. lactis* was induced with different concentrations of nisin (0, 1, 2.5, 5, 10, 20, and 40 ng/mL) for 9 h and at different times (0, 3, 6, 9, 12, 15, 18, 21, and 24 h) with 10 ng/mL nisin. The cultures were centrifuged at 7500 g and 4 °C for 15 min with the cell pellets discarded. To inhibit proteolysis in the supernatant, 1 mM phenylmethylsulfonylfluoride (PMSF; Thermo Fisher Scientific, Waltham, MA, USA) and 10 mM dithiothreitol (DTT; Thermo Fisher Scientific, Waltham, MA, USA) were added. Loading buffer (10 μL, 5×; Beijing Solarbio Science & Technology Co., Ltd., Beijing, China) was added to 40 μL of the supernatant, which was then denatured at 100 °C for 8 min. Western blot was performed on 20 μL of the supernatants.

### 2.4. Purification of Recombinant gEGF Protein from Lactococcus Lactis

Protein samples were prepared from 300 mL of the recombinant *L. lactis* cultures after 6 h of induction at 30 °C without vibration. Supernatants obtained by centrifugation at 7500 g for 20 min at 4 °C were subjected to precipitation with ammonium sulfate (80%, *w*/*v*; Sinopharm Chemical Reagent Co. Ltd., Beijing, China) The sample was incubated at 4 °C with automatic stirring for 2 h and then centrifuged at 12,000 g for 30 min at 4 °C. The resultant pellet and floating materials were harvested and dissolved in a binding buffer comprising 500 mM NaCl, 20 mM Na3PO4, and 30 mM imidazole at pH 7.4 and dialyzed overnight using a 1.0-kDa cut-off membrane against the same buffer at 4 °C. The supernatant was centrifuged at 10,000× *g* for 15 min and passed through a 0.45-μm filter (Merck Millipore Ltd., Billerica, MA, USA) to remove insoluble debris. The supernatant was loaded into a HisTrap HP column (GE Healthcare Co., Ltd., Buckinghamshire, UK) pre-equilibrated with binding buffer and shaken gently at 4 °C for 60 min. The column was then washed with the native wash buffer (500 mM NaCl, 20 mM Na3PO4, and 60 mM imidazole at pH 7.4). Finally, the bound protein was eluted with native elution buffer (500 mM NaCl, 20 mM Na3PO4, and 500 mM imidazole at pH 7.4) and analyzed using Tricine-SDS-PAGE. The purified protein was then dialyzed against the phosphate-buffered saline (PBS). The concentration of the purified protein was determined using a bicinchoninic acid (BCA) protein assay kit (Thermo Fisher Scientific, Waltham, MA, USA).

### 2.5. In Vitro Experiment

The UMNSAH/DF-1 cells (ATCC-CRL-12203), a chicken embryo fibroblast line (BeNa Culture Collection Co., Ltd., Beijing, China), were cultured for 24 h in Dulbecco’s modified Eagle’s medium (DMEM; Thermo Fisher Scientific, Waltham, MA, USA) containing 10% fetal bovine serum (Thermo Fisher Scientific, Waltham, MA, USA) until they attained a full monolayer and then diluted at 1:10. Next, 100 μL of the diluted cell culture was added into each well of a 96-well Cell Culture Cluster (Corning Inc., Shanghai, China) and cultured for 12 h in DMEM at 37 °C to achieve 60% of the cells of the full monolayer and then starved for 12 h in DMEM without fetal bovine serum at 37 °C. The cell cultures were washed with 100 μL PBS before being treated with 100 μL DMEM containing gEGF (1, 5, 10, and 20 μg/mL) for 12 h, while the control was treated with 100 μL DMEM without gEGF. Finally, 10 μL Cell Counting Kit-8 (Dojindo Molecular Technologies, Inc., Rockville, MD, USA) was added to the cell cultures and incubated for 1 h. The OD_450_ of the cultures was measured using a MULTISKAN MK3 (Thermo Electron Corporation, Waltham, MA, USA).

### 2.6. In Vivo Animal Experiment

The study was conducted according to the guidelines of the Care and Use of Laboratory Animals Monitoring Committee of Hubei Province, China, and approved by the Committee on the Ethics of Animal Experiments at the College of Veterinary Medicine, Huazhong Agricultural University (protocol code 42000400002483 in November, 2019). A total of 63,817 hybrid broilers (Single Cross, crosses of Rose 308 × Hy-Line Brown layers, Wuhan CP Co., Ltd., Wuhan, China) 5 days old were evenly divided into three groups (each group of 21 chicks) by simple randomization, each containing 3 replications of 7 broilers (each replication was treated as one experimental unit) and allocated to three different treatments for 15 days of: (1) M17 medium (the control group); (2) the supernatant of the LL-pNZ8149 fermentation product (the P-LL group); and (3) the supernatant of the LL-pNZ8149-gEGF fermentation product (the gEGF group). The temperature of the chicken coop was maintained at approximately 33 °C before the chickens were 7 days old and then gradually reduced to 23 °C when the chickens were between 7 and 20 days old. The temperature was maintained at 23 °C thereafter. For the first 3 days, 24 h of light was provided. The light period was reduced to 18 h from 4 to 12 days and to 16 h from 13 to 20 days. The investigators were blinded to group allocation during the entire experiments and analyses. The initial average body weight of each group was 91.43 ± 2.65 g, 92.00 ± 1.98 g, and 91.42 ± 1.51 g, respectively (data presented as mean ± standard deviation). The supernatant was prepared by the centrifugation of the fermentation product at 10,000× *g* for 15 min and discarding the bacterial pellet. Thereafter, all broilers in three groups were orally gavaged with 1 mL each of their treatment agents once a day with the treatment of 2.67 μg gEGF for the gEGF group. All chickens were separately fed in a metal cage with enough corn-soybean meal and were provided ad libitum access to water and feed for the 15-day experimental period. Chickens were reared in an environmentally regulated nursery house at Huazhong Agricultural University. The list of the feed formulation and nutrient composition were provided in Table 1.

Feed intakes and body weights were recorded every 7 days. All of the broilers were healthy throughout the entire trial. On day 15, 3 broilers from each cage were randomly selected (9 broilers from 3 cages for each treatment) for the collection of samples to evaluate the effect of gEGF on their immune function and intestinal development. Blood was collected from the wing vein of all randomly selected broilers before they were anaesthetized according to O’Kane’s method [27] and sacrificed by exsanguinations for the sample collection of intestinal tissues and immune organs. Care and maintenance of all animals were in line with the Guide for the Care and Use of Laboratory Animals Monitoring Committee of Hubei Province, China.

Blood samples (around 3 mL each tube, 2 tubes each chick) were taken from the wing vein, collected into vacuum blood collection tubes. The collected blood samples were centrifuged at 260 g and 4 °C to obtain the cell pellets. Thymus, spleen, and bursa of Fabricius were collected and immediately weighed from the broilers after they were euthanized. The spleen index, Bursa of Fabricius index and thymus index of broilers were calculated as follows: (1) Spleen index (mg/g) = Spleen weight (mg)/body weight (g), (2) Bursa of Fabricius index (mg/g) = Bursa of Fabricius (mg)/body weight (g), and (3) Thymus index (mg/g) = Thymus weight (mg)/body weight (g). The intestinal tract samples of the duodenum, jejunum, and ileum were cut to open longitudinally, flushed with precooled PBS, and blotted to remove excess fluid. Dissected samples were fixed with 4% paraformaldehyde (Biosharp Co., Ltd., Hefei, China) for paraffin sectioning to evaluate the villus-crypt morphology. A 5 cm section of each tissue from the intestinal tract was scraped with a scalpel to obtain mucosal samples. Mucosal scrapings were placed on pre-weighed cryovials, weighed, and frozen in liquid nitrogen for identification of mucosal sIgA. Intestinal samples were embedded in paraffin, sectioned (3-μm thickness), stained with hematoxylin and eosin for panoramic scanning, and imaged with a Panoramic MIDI slide scanner (3D HISTECH Co., Ltd., Budapest, Hungary). Villus height and crypt depth were measured from 8 appearance-intact villi with Image-pro plus 6.0 (Media Cybernetics, Inc., Bethesda, MD, USA). Villus height was represented by the distance from the villus tip to the crypt opening, and crypt depth was determined from the crypt opening to the base. Frozen mucosa samples were homogenized on ice in saline using a Pro 200 homogenizer (PRO Scientific Co., Ltd., Oxford, CT, USA). Serum IgA and IgG and small intestinal mucosal sIgA were measured using ELISA kits (Shanghai Bogoo Biotechnology Co., Ltd., Shanghai, China).

### 2.7. Statistical Analysis

The in vivo experimental model used was a completely randomized design with 3 treatments, each containing 3 replications each of 7 broilers. Each replication containing 7 broilers was treated as one experimental unit. All data were analyzed using the statistical program SPSS 19 (IBM Corp., Armonk, NY, USA). The one-way ANOVA was performed using Fisher’s protected LSD test for multiple comparisons. Results were regarded as significant when *p* < 0.05.

## 3. Results

### 3.1. Expression of gEGF Protein in a Bacterial Culture Supernatant

**Figure 1:**
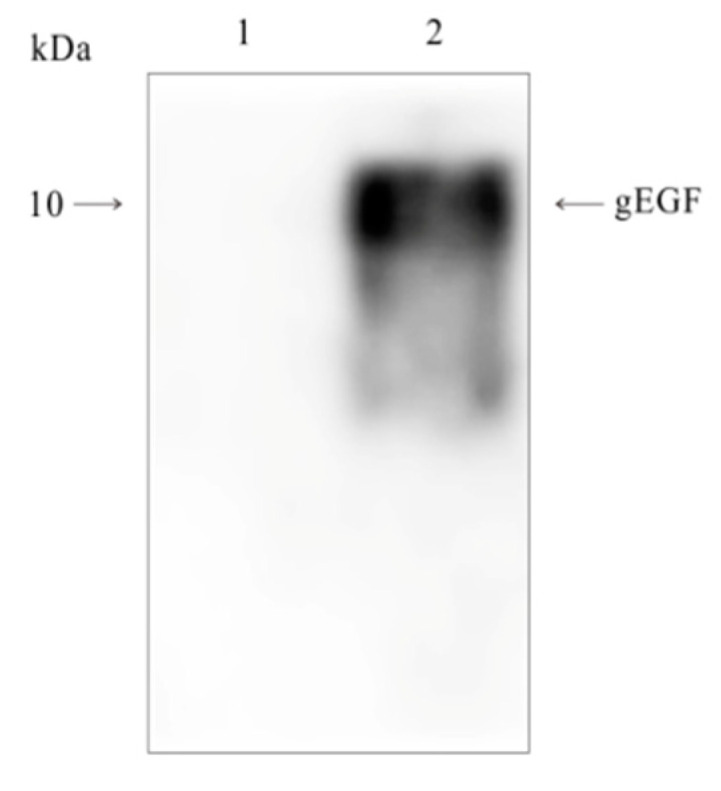
Hybridization of the His-tag monoclonal antibody with the gEGF protein produced by the *Lactococcus lactis* recombinant strain. Lanes 1 and 2: 20 μL/well of the supernatants from the LL-pNZ8149 and the LL-pNZ8149-gEGF fermentation products, respectively. The recombinant gEGF is marked by an arrowhead.

Figure 1 showed that the protein from the LL-pNZ8149-gEGF fermentation product hybridized with the His-tag monoclonal antibody, indicating the presence of the target protein.

**Figure 2:**
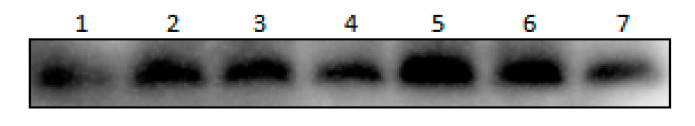
Analysis of gEGF expression by LL-pNZ8149-gEGF induced with different concentrations of nisin for 9 h when the optical density of 600 nm (OD_600_) of the bacterial cultures reached 0.4. Lanes 1–7: the 20 μL/well of supernatant from the induced cultures of the LL-pNZ8149-gEGF with nisin of 0, 1, 2.5, 5, 10, 20, and 40 ng/mL, respectively.

**Figure 3:**
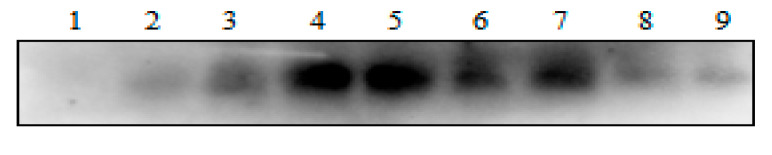
Analysis of gEGF expression by LL-pNZ8149-gEGF induced with nisin (10 ng/mL) using different induction times when the optical density of 600 nm (OD_600_) of the bacterial cultures reached 0.4. Lanes 1–9: the 20 μL/well of the supernatant from the induced cultures of the LL-pNZ8149-gEGF for 0, 3, 6, 9, 12, 15, 18, 21, and 24 h, respectively.

The expressions of gEGF in the supernatant of the LL-pNZ8149-gEGF fermentation product induced for 9 h with different concentrations of nisin (0, 1, 2.5, 5, 10, 20, and 40 ng/mL) and different induction times (0, 3, 6, 9, 12, 15, 18, 21, and 24 h) with 10 ng/mL nisin were shown in Figure 2 and Figure 3, respectively. As nisin concentrations increased from 0 to 40 ng/mL, the expression of gEGF initially increased (*p* < 0.05) but started to decrease at 10 ng/mL. A similar pattern was observed as induction time increased from 0 to 24 h, showing that the expression of gEGF initially increased and then started to decrease at 12 h.

**Figure 4:**
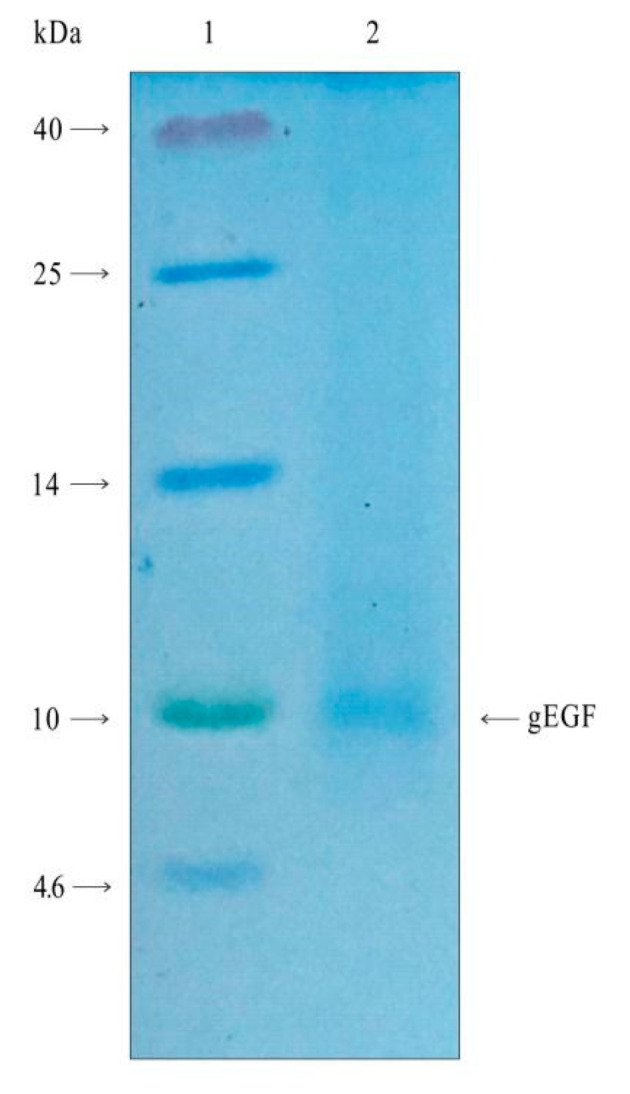
Tricine-SDS-PAGE analysis of the purified gEGF protein. Lane 1: Spectra Multicolor Low Range Protein Ladder (kDa). Lane 2: 20 μL/well of protein purified from the supernatant of 300 mL LL-pNZ8149-gEGF cultures. The recombinant gEGF is marked with an arrowhead at 7 kDa.

**Figure 5:**
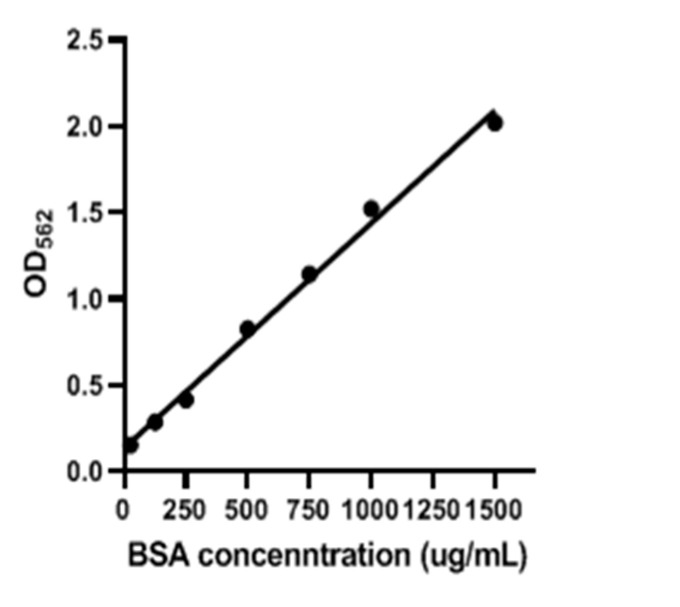
The standard curve for bovine serum albumin (BSA) based on the bicinchoninic acid (BCA) protein assay analysis.

The results of an SDS-PAGE analysis of the purified protein of gEGF from the supernatant of the LL-pNZ8149-gEGF fermentation product were shown in Figure 4. The band near 7 kDa corresponded to the computed molecular weight (MW) of gEGF. The standard curve of bovine serum albumin obtained by the BCA protein assay kit was given in Figure 5. By calculating the standard curve, the protein concentration of 5 mL purified protein was 0.16 mg/mL, and the concentration of gEGF in the supernatant of LL-pNZ8149-gEGF fermentation product was 2.67 μg/mL.

### 3.2. gEGF Promotes UMNSAH/DF-1 Cell Proliferation

**Figure 6:**
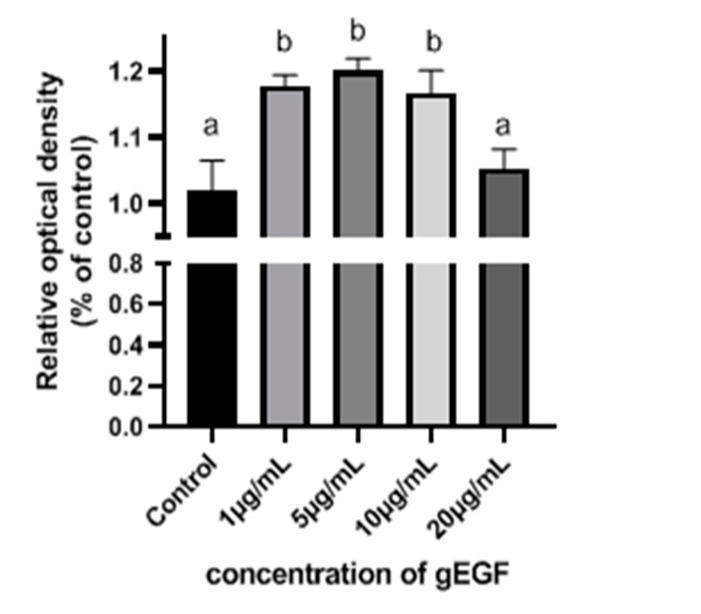
Effect of gEGF of various concentrations on UMNSAH/DF-1 cell proliferation. Values are given as mean ± SD (*n* = 5). Bars with different letters (a and b) show the values with a significant difference (*p* < 0.05).

The effects of gEGF on UMNSAH/DF-1 cell proliferation were shown in Figure 6. The UMNSAH/DF-1 cell proliferation was significantly enhanced by the treatments of gEGF with concentrations of 1, 5, and 10 μg/mL, while the strongest effect was observed at 5 μg/mL of gEGF.

### 3.3. gEGF Promotes the Growth Performance of Broilers

In comparison to the control and the P-LL groups, the average body weight (ABW), the average daily feed intake (ADFI), and the average daily gain (ADG) in the gEGF group significantly increased after 1 and 2 weeks (Table 2). The gain:feed ratio in both the P-LL and the gEGF groups increased significantly in comparison to the control group in the first week (Table 2), though no significant difference was observed between these two experimental groups (Table 2). In two weeks, the gain:feed ratio of the gEGF group significantly increased compared with those of the control and the P-LL groups (Table 2).

### 3.4. gEGF Improves the Immune Function of Broilers

Table 3 showed the effects of gEGF on the indices of different immune organs from the treated broilers. Compared with those of the control and the P-LL groups, the indices of spleen and thymus gland in the gEGF group increased significantly in two weeks. Furthermore, the BF Index of the gEGF group was significantly higher than that of the P-LL group (Table 3) but not of the control group (Table 3).

The effects of gEGF on serum and mucosa immunoglobulins of the treated broilers after two weeks were shown in Table 4. The concentrations of serum IgA and IgG in the gEGF group were significantly higher than those in the control and the P-LL groups, while the duodenum mucosal sIgA increased significantly in the gEGF group compared to those in the control and the P-LL groups.

### 3.5. gEGF Promotes the Intestinal Development of Broilers

The effects of gEGF on the mucosal morphological traits of the small intestines of broilers after two weeks of treatment were shown in Table 5. The villus heights of the duodenum, jejunum, and ileum in the gEGF group were significantly higher than those in the control and the P-LL groups. The crypt depth of the jejunum in the gEGF group was significantly deeper than those of the other two groups, while the crypt depth of the duodenum in the gEGF group were significantly shallower than those in the P-LL group (Table 4) but not in the control group (Table 5). Furthermore, the gEGF treatment significantly increased the ratio of villus height to crypt depth (VH/CD) of the duodenum, but not those of the jejunum and the ileum.

## 4. Discussion

The epidermal growth factor (EGF) is a 53–amino acid single-chain polypeptide found in mammalian colostrum and milk [28] with low yields generated using previous extraction methods [15,16,19,29]. To date, EGF is mass produced by utilizing a variety of expression systems. However, studies have revealed that different systems yield remarkably different expression levels of EGF and, depending on the conditions, the levels of EGF vary greatly, even utilizing the same system [13,15,16,17,18,19,30]. Studies have shown that the protein production in nisin-induced *Lactococcus lactis* expression systems could be increased by optimizing various experimental factors [31]. In the present study, gEGF was expressed with a nisin-induced *L. lactis* expression system with the optimal nisin concentration and induction time identified for yield of protein. Our results showed that both the concentration of nisin and induction time could significantly affect the expression level of gEGF. As the concentration of nisin increased, the gEGF yield increased initially, followed by a decrease starting at 10 ng/mL. While a threshold concentration of nisin is required for the complete induction, excess concentrations could inhibit the growth of the host strain, probably due to the innate antibacterial properties. Furthermore, as the induction time increased, the expression level of EGF increased gradually then decreased, starting at 12 h, perhaps due to the excessive lactate accumulation disrupting the cell metabolism during cell growth [32,33]. The concentration of gEGF in the supernatant was 2.67 μg/mL, which was comparable to or even higher than those reported previously [15,17,19,26].

Our results showed that gEGF promotes UMNSAH/DF-1 cell proliferation. Despite the minor differences in the amino acid sequences of EGF from different species, the shared structure of the EGF receptor (EGFR) ensures that EGF is not highly species-specific. For example, human epidermal growth factor (hEGF) has been shown to function in rats [34], mouse epidermal growth factor (mEGF) in pigs [35], and pEGF in chickens [24]. However, some anomalies reported to show that the binding ability of mEGF to chicken EGFR was 100 times lower than that to human EGFR, while the binding ability of TGF-alpha to chicken EGFR was equal to or higher than that to human EGFR [36]. These results illustrated the differences in the binding ability of EGF compared to that of EGFR in different species [37], indicating that the EGF of a given species may show different effects on a different species. Studies have shown that the proliferation of human dermal fibroblasts is promoted by hEGF [2]. Therefore, in the current study, a chicken embryo fibroblast line (i.e., UMNSAH/DF-1) was used to investigate the biological activities of gEGF in vitro. Our results showed that gEGF could significantly promote the proliferation of UMNSAH/DF-1 cells. Moreover, the ability to promote cell proliferation was increased initially by the treatment of gEGF with a concentration lower than 5 μg/mL then decreased at 20 μg/mL, indicating that the proliferation-promoting effect of gEGF was concentration-dependent.

Our study evaluated the growth performance of the broilers by measuring the average body weight, the average daily gain, the daily feed intake, and the gain:feed ratio. Most indices of growth performance were significantly improved during the first week of gEGF treatment. Our results showed that the gain:feed ratio increased in both the P-LL and gEGF groups compared with that in the control group within the first week, suggesting that some components of the fermentation product of LL-pNZ8149 affected the growth performance. However, the gain:feed ratio of the gEGF group was significantly higher than that of the other two groups following two weeks of gEGF treatment, indicating that gEGF was an important factor in the growth of broilers. These results suggested that gEGF significantly improved the growth performance of broilers via increasing the gain:feed ratio. Kim et al. showed that mEGF demonstrated beneficial effects on the growth performance of broiler chickens prior to the infection by *Eimeria*, while enhancing the expression of the nutrient transporter gene *xCT1* [24]. Bedford et al. found that the supernatant of the EGF-LL fermentation product increased the levels of sucrase and alkaline phosphatase in 8 days of treatment [19]. Furthermore, Wang et al. reported that the sucrase in 3 intestinal segments, the aminopeptidase A in the duodenum and jejunum, and the aminopeptidase N and dipeptidase Ⅳ in the duodenum were significantly higher in the group treated with LL-pEGF than in the control group [2]. These results suggest that the enhanced expression of nutrient transporters and increased levels of digestive enzymes caused by EGF may have contributed to the improved growth performance of broilers and early-weaned piglets. However, Bedford et al. showed that the gain:feed ratio of early-weaned piglets did not increase following two weeks of treatment with the supernatant of recombinant bacteria [19]. Therefore, further studies are necessary to investigate whether the growth-promoting effect of gEGF on livestock and poultry is transient. Overall, our results suggest that gEGF may be utilized to enhance the growth performance in broiler chickens.

The effect of gEGF on the immune function of broilers was evaluated by measuring the immune organ indices, serum levels of IgA and IgG, and the level of the intestinal mucosal sIgA. Studies investigating the effects of EGF on the immune functions are sparse. Dietary supplementation of recombinant pEGF was shown to effectively increase the level of IgA in the jejunum mucosa of weaned piglets [38,39,40]. Furthermore, the level of serum IgA in weaned piglets increased significantly after 18 days of treatment with pEGF, while the same treatment significantly increased the level of IgA in the jejunum mucosa after 28 days [20]. In our study, serum IgA increased significantly in two weeks of gEGF treatment, which was similar to the effect of pEGF in piglets [20]. However, the level of sIgA did not increase in the mucosa of either the jejunum or the ileum, probably due to the degradation of gEGF along the gastrointestinal tract as degradation rates of EGF in the small intestinal lumen of weaned pigs has been shown to increase from the proximal to the mid and distal regions [41], resulting in relatively high levels of gEGF in the proximal intestinal tract. Furthermore, our results have shown that the thymus and spleen indices increased significantly in broilers treated with gEGF in two weeks, indicating that the immune function was improved compared to the phenotype of the other two groups of chicken. Overall, these results suggest that the gEGF could significantly improve immune function in broilers.

The effect of gEGF on the intestinal development of broilers was evaluated by measuring villus height, crypt depth, and the VH/CD ratio in the mucosa of the duodenum, jejunum, and ileum. Intestinal villus with a greater height has a larger area for nutrient absorption, leading to the enhanced absorption, while shallower crypts lead to higher maturation rates of intestinal epithelial cells and better absorption. The VH/CD value could reflect the function of the small intestine, with higher values implying higher nutrient absorption in the intestines [42]. Our results clearly showed that gEGF promoted the development of the intestinal villus in the duodenum, jejunum, and ileum following two weeks of gEGF treatment, while the results of crypt depth showed that gEGF promoted the development only in the duodenum. Therefore, the gEGF showed a pronounced effect on the villus, in contrast to the previous results reporting that EGF showed greater effects on the crypt [43]. Moreover, gEGF was strongly associated with the development of the duodenum, probably also due to the varied degradation rates of gEGF within different regions of the intestines [40].

The EGF has been reported to promote growth performance, improve immune function, and stimulate intestinal development or repair in animals [2,19,20,44]. Wang et al. showed that early-weaned piglets reared with LL-pEGF significantly increased the final average body weight and average daily gain in 14 days [44]. Bedford et al. reported that the supernatant of recombinant *Lactococcus lactis* could promote growth performance in early-weaned piglets, while the fermentation product with the expression of EGF did not show the enhancement of growth performance, possibly due to the bacterial overload [19]. To date, studies have focused on EGF in mammals [20,45,46], and there are no studies on EGF and its biological activities in chickens. Therefore, it is practically important to investigate the gEGF and its biological activities in relation to growth performance, immune function, and the intestinal development of broilers.

Our results using gEGF showed that this therapy significantly promoted the growth performance of broiler chickens via increased food intake and weight gain, improved nutrient absorption, and enhanced intestinal and immune functions. Therefore, we recommend that gEGF be used as an alternative growth promotant because the gEGF exhibited non-toxic side effects and no antibacterial effect that could influence the natural intestinal microbiota of consumers, ultimately avoiding the risks associated with chicken and food safety. We note that the limitation of this study was that the chickens were grown for only two weeks. Further studies with a longer growing time (for 6 weeks) are necessary to further confirm the effects of gEGF on the growth performance observed in the current study and to see whether this treatment could ultimately yield larger, healthier birds. It is worth noting how to increase the production of gEGF. At present we have a preliminary progress. We are optimizing the production process and testing with 14,000 817 hybrid broilers (Single Cross, crosses of Rose 308 × Hy-Line Brown layers, Wuhan CP Co., Ltd., Wuhan, China).

## 5. Conclusions

In this work, we construct a recombinant strain of gEGF-expressing *Lactococcus lactis* produced the secretory form of gEGF and the expression level of gEGF was determined by the concentration of the inducer (i.e., nisin) and the induction time. The optimal concentration of the inducer (i.e., nisin) is 10 ug/mL and the optimal induction time is 12 h, and the concentration of gEGF in the supernatant of LL-pNZ8149-gEGF fermentation product was 2.67 μg/mL. In vitro experiment results showed that gEGF could stimulate the proliferation of chicken embryo fibroblast cells, and in vivo experiment results showed that feeding gEGF for two weeks can enhance the growth performance, promote the intestinal development, and improve the immune functions of broiler chickens. This study provides the scientific evidence to support the application of gEGF as an alternative growth promotant in commercial broiler productivity.

## Figures and Tables

**Table 1:** Composition of experimental diets. Diet: zinc, 60 mg; iron, 95 mg; copper, 10 mg; iodine, 0.35 mg; selenium, 0.3 mg; manganese, 80 mg; vitamin A, 12,000 IU; vitamin D3, 2750 IU; vitamin E, 30 IU; vitamin K3, 2 mg; vitamin B12, 12 μg; riboflavin, 6 mg; nicotinic acid, 40 mg; pantothenic acid, 12 mg; pyridoxine, 3 mg; biotin, 0.2 mg.

Items	Content (%)
Ingredients (%)	
Corn, yellow	58.10
Soybean meal, dehulled (CP, 48%)	30
Fish meal	4.94
Soybean oil	3.0
Calcium bicarbonate	1.4
Limestone	1.4
Salt	0.25
L-Lysine-HCl	0.03
DL-methionine	0.2
Choline chloride	0.13
Antioxidant	0.05
Mineral and vitamin premix (supplied per kilogram)	0.05
Nutrient content (%)	
Crude protein (%)	21
Crude fiber (%)	4.95
Calcium (%)	0.95
Phosphorus (%)	0.62
Lysine (%)	1.30
Methionine (%)	0.55
Met + Cys (%)	0.90
ME (kcal/kg)	3100

**Table 2:** Effects of gEGF on the growth performance and feed efficiency of broilers. SEM: Standard Error of Mean. Data represented the mean of 3 replications per treatment with 7 broilers per replication (*n* = 3). Means within a row without superscripts (a or b) were of no significant difference (*p* > 0.05). Means within a row with different superscripts (a or b) differed significantly (*p* < 0.05). IABW: initial average body weight; ABW: average body weight; ADFI: average daily feed intake; ADG: average daily gain.

Item	Time	Treatment			SEM	*p*-Value
		Control	P-LL	gEGF		
IABW (g)		91.43	92.00	91.42	1.79	0.930
ABW (g)	Week 1	210.47 ^a^	225.52 ^a^	238.62 ^b^	5.62	0.018
	Week 2	395.14 ^a^	390.19 ^a^	442.10 ^b^	12.46	0.006
ADFI (g)	Week 1	30.43 ^a^	31.12 ^a^	33.57 ^b^	0.71	0.011
	Week 2	33.12 ^a^	32.65 ^a^	36.40 ^b^	1.02	0.020
ADG (g/d)	Week 1	17.01 ^a^	19.10 ^a^	21.03 ^b^	0.67	0.007
	Week 2	26.59 ^a^	26.08 ^a^	29.87 ^b^	1.32	0.018
Gain:feed (g/g)	Week 1	0.56 ^a^	0.61 ^b^	0.63 ^b^	0.17	0.016
	Week 2	0.80 ^a^	0.80 ^a^	0.82 ^b^	0.06	0.027

**Table 3:** Effects of gEGF on the indices of immune organs of treated broilers in two weeks of treatment. SEM: Standard Error of Mean; BF: bursa of Fabricius; TG: Thymus gland. Data represented mean of 3 replications per treatment with 3 broilers per replication (*n* = 3). Means within a row without superscripts (a or b) were of no significant difference (*p* > 0.05). Means within a row with different superscripts (a or b) differed significantly (*p* < 0.05).

Item	Treatment			SEM	*p*-Value
	Control	P-LL	gEGF		
BW (g)	397.11 ^a^	395.34 ^a^	446.67 ^b^	4.29	<0.001
Spleen weight (g)	0.38 ^a^	0.38 ^a^	0.57 ^b^	0.28	0.001
Index of spleen (spleen weight (mg)/BW (g))	0.95 ^a^	0.97 ^a^	1.28 ^b^	0.06	0.002
BF weight (g)	1.43 ^a^	1.31 ^a^	1.69 ^b^	0.62	0.002
Index of BF (BF weight (mg)/BW (g))	3.59 ^a,b^	3.30 ^a^	3.78 ^b^	0.14	0.040
TG weight (g)	2.18 ^a^	2.24 ^a^	2.86 ^b^	0.77	<0.001
Index of TG(TG weight (mg)/BW (g))	5.50 ^a^	5.66 ^a^	6.40 ^b^	0.20	0.008

**Table 4:** Effects of gEGF on serum and mucosal immunoglobulins of treated broilers in two weeks of treatment. SEM: Standard Error of Mean. Data represented mean of 3 replications per treatment with 3 broilers per replication (*n* = 3). Means within a row without superscripts (a or b) were of no significant difference (*p* > 0.05). Means within a row with different superscripts (a or b) differed significantly (*p* < 0.05).

Item		Treatment			SEM	*p*-Value
		Control	P-LL	gEGF		
Serum	IgA (mg/mL)	0.27 ^a^	0.23 ^a^	0.30 ^b^	0.01	0.004
	IgG (mg/mL)	4.07 ^a^	3.96 ^a^	5.09 ^b^	0.34	0.010
Mucosal sIgA (ng/mg)	Duodenum	197.20 ^a^	193.80 ^a^	371.34 ^b^	8.13	<0.001
	Jejunum	226.65	247.90	219.22	26.27	0.559
	Ileum	619.85	603.26	640.77	26.82	0.428

**Table 5:** Effects of gEGF on the mucosal morphological traits of the small intestines of treated broilers after two weeks of treatment. SEM: Standard Error of Mean. Data represented mean of 3 replications per treatment with 3 broilers per replication (*n* = 3). Means within a row without superscripts (a or b) were of no significant difference (*p* > 0.05). Means within a row with different superscripts (a or b) differed significantly (*p* < 0.05).

Item	Treatment			SEM	*p*-Value
	Control	P-LL	gEGF		
Duodenum					
Villus height (μm)	1116.11 ^a^	1210.01 ^a^	1333.72 ^b^	41.85	0.006
Crypt depth (μm)	97.17 ^a,b^	97.08 ^a^	84.79 ^b^	6.32	0.016
VH/CD	11.59 ^a^	12.47 ^a^	15.73 ^b^	0.64	0.001
Jejunum					
Villus height (μm)	763.91 ^a^	757.41 ^a^	951.30 ^b^	16.90	<0.001
Crypt depth (μm)	97.63 ^a^	100.03 ^a^	130.51 ^b^	7.31	0.007
VH/CD	7.87	7.62	7.29	0.47	0.504
Ileum					
Villus height (μm)	652.11 ^a^	648.67 ^a^	695.15 ^b^	13.72	0.026
Crypt depth (μm)	82.94	85.01	88.47	6.07	0.673
VH/CD	7.86	7.72	7.87	0.50	0.949

## Data Availability

The data used to support the findings of this study are available from the corresponding author upon request.

## Abbreviations

gEGF: *gallus* epidermal growth factor; LL-pNZ8149: *Lactococcus lactis* transformed with plasmid pNZ8149; LL-pNZ8149-gEGF: *Lactococcus lactis* that secretes gEGF; P-LL: treatment group fed supernatant of LL-pNZ8149 fermentation product; EGFR: epidermal growth factor receptor; pEGF: porcine epidermal growth factor; mEGF: mouse epidermal growth factor; hEGF: human epidermal growth factor; IABW: initial average body weight; ABW: average body weight; BW: body weight; ADFI: average daily feed intake; ADG: average daily gain.

## Appendix A

Sequence of fusion gene fragment of gEGF and SPusp45: CCATGGGTATGAAAAAAAAGATTATCTCAGCTATTTTAATGTCTACAGTGATACTTTCTGCTGCAGCCCCGTTGTCAGGTGTTTACGCTGGTGATTCAATCGGTTGTCCACCAGCTTACGATTCATACTGTCTTCACGGTGGTGTTTGTAACTACGTTTCAGATCTTCAAGATTACGCTTGTAACTGTGTTACTGGTTACGTTGGTGAACGTTGTCAATTCTCAGATCTTGAATGGTGGGAACAACAACACGCTGAACGTCACCACCACCACCACCACTAGGAGCTC

Note: letters in blue, red, and green are sequences of SPusp45, gEGF, and 6 × His-tag, respectively.
